# Supplementary material for: Effect of cystic fibrosis transmembrane conductance regulator modulators and dedicated cystic fibrosis gastrointestinal clinic visits on the incidence of distal intestinal obstructive syndrome in persons with cystic fibrosis
Source: PLoS One. 2025 Jul 28;20(7):e0328015. doi: 10.1371/journal.pone.0328015 (PMC12303333; doi:10.1371/journal.pone.0328015)
Supplement: S1 Data — (DOCX) [file pone.0328015.s001.docx]

**Supplementary data 1.** Characteristics of patients seen in cystic fibrosis-gastrointestinal clinic for comparison of three years before and after cystic fibrosis-gastrointestinal clinic

| Characteristics | N=59 |
| --- | --- |
| Sex, female | 31 (52.5%) |
| Age, year, Mean (SD) | 41.2 (10.0) |
| Race  White  Black  Asian/Pacific Islander  Missing | 58 (98.3%)  0 (0.0%)  0 (0.0%)  1 (1.7%) |
| Ethnicity  Non-Hispanic  Hispanic  Missing | 38 (64.4%)  0 (0.0%)  21 (35.6%) |
| CFTR gene mutation  - F508 deletion homozygous  - F508 deletion heterozygous  - Others | 32 (54.2%)  24 (40.7%)  3 (5.1%) |
| CFTR modulator use | 46 (78.0%) |
| Exocrine pancreatic insufficiency | 54 (91.5%) |
| Cystic fibrosis-related diabetes | 33 (55.9%) |
| Lung transplant | 11 (18.6%) |
| Gastroparesis confirmed with four-hour gastric emptying study | 12 (20.3%) |
| Cirrhosis, seen by liver clinic | 2 (3.4%) |
| Prior history of DIOS | 36 (61.0%) |

Abbreviation: CFTR cystic fibrosis transmembrane conductance regulator; DIOS distal intestinal obstruction syndrome
