# Supplementary material for: Effect of cystic fibrosis transmembrane conductance regulator modulators and dedicated cystic fibrosis gastrointestinal clinic visits on the incidence of distal intestinal obstructive syndrome in persons with cystic fibrosis
Source: PLoS One. 2025 Jul 28;20(7):e0328015. doi: 10.1371/journal.pone.0328015 (PMC12303333; doi:10.1371/journal.pone.0328015)
Supplement: S2 Data — (DOCX) [file pone.0328015.s002.docx]

**Supplementary data 2.** Characteristics of patients seen in cystic fibrosis-gastrointestinal clinic for comparison of pre- and post-cystic fibrosis transmembrane conductance regulator (CFTR) modulator initiation

| Characteristics | N=125 |
| --- | --- |
| Sex, female | 64 (51.2%) |
| Race  White  Black  Asian/Pacific Islander  Missing | 121 (96.8%)  1 (0.8%)  1 (0.8%)  2 (1.6%) |
| Ethnicity  Non-Hispanic  Hispanic  Missing | 88 (70.4%)  1 (0.8%)  36 (28.8%) |
| CFTR gene mutation  - F508 deletion homozygous  - F508 deletion heterozygous  - Others | 69 (55.2%)  51 (40.8%)  5 (4.0%) |
| Exocrine pancreatic insufficiency | 114 (91.2%) |
| CF-related diabetes | 62 (49.6%) |
| Lung transplant | 11 (8.8%) |
| Gastroparesis confirmed with four-hour gastric emptying study | 5 (4.0%) |
| Cirrhosis, seen by liver clinic | 7 (5.6%) |
| Prior history of DIOS | 61 (48.8%) |

Abbreviation: CFTR cystic fibrosis transmembrane conductance regulator; DIOS distal intestinal obstruction syndrome
