# Supplementary material for: Effect of cystic fibrosis transmembrane conductance regulator modulators and dedicated cystic fibrosis gastrointestinal clinic visits on the incidence of distal intestinal obstructive syndrome in persons with cystic fibrosis
Source: PLoS One. 2025 Jul 28;20(7):e0328015. doi: 10.1371/journal.pone.0328015 (PMC12303333; doi:10.1371/journal.pone.0328015)
Supplement: S3 Data — (DOCX) [file pone.0328015.s003.docx]

**Supplementary data 3.** Changes in number of admissions and ED visits before and after CFTR modulator initiation by types of CFTR modulators

| CFTR type | N | ED DIOS diff (pre-post) | Admission DIOS diff (pre-post) |
| --- | --- | --- | --- |
| Lumacaftor/ivacaftor | 49 | 1 decrease, 4 increase | 5 decrease, 13 increase |
| Elexacaftor/tezacaftor/ivacaftor | 47 | 3 decrease, 1 increase | 5 decrease, 4 increase |
| Tezacaftor/ivacaftor | 18 | 1 decrease, 1 increase | 2 decrease, 5 increase |
| Ivacaftor | 8 | 1 increase | 1 increase |
| Missing data on CFTR type | 3 | All no differences | All no differences |

Abbreviation: CFTR cystic fibrosis transmembrane conductance regulator; DIOS distal intestinal obstruction syndrome
